# Antiphotoaging effects of a group of antioxidant peptides through downregulating matrix metalloproteinases and inflammation factors

**DOI:** 10.3389/fcell.2025.1649391

**Published:** 2025-11-27

**Authors:** Yichao Huang, Juxingsi Song, Zhengbang Wang, Shaoqian Zhu, Yanan Hu, Xinyue Gan, Sai Luo, Qian He, Liming Zhang, Qianqian Wang

**Affiliations:** 1 Naval Special Medical Center, Naval Medical University, Shanghai, China; 2 College of Basic Medical Sciences, Naval Medical University, Shanghai, China; 3 The Third Affiliated Hospital, Naval Medical University, Shanghai, China

**Keywords:** antioxidant peptides, anti-photoaging, matrix metalloproteinases, ultraviolet B, inflammation factors

## Abstract

Excessive ultraviolet B (UVB) exposure is the leading environmental contributor to the development of skin aging, also referred to as photoaging. We previously obtained a group of modified antioxidant peptides (WP5, LW5 and YY6), which were derived from marine organisms, through a single amino acid substitution, and demonstrated that the antioxidant peptides exhibited apparent protective effects against UVB-induced oxidative damage in human keratinocyte cells. Nonetheless, it remains uncertain whether they can alleviate skin photoaging caused by UVB. This research aims to investigate the anti-photoaging effects of the antioxidant peptides *in vitro* and *in vivo* and the underlying molecular mechanisms. We found that the antioxidant peptides significantly alleviated the senescence of HDF-a cells induced by UVB and suppressed the expression of matrix metalloproteinases (MMPs) and the degradation of collagen I, and the mitogen-activated protein kinase (MAPK) pathways, particularly the p38 MAPK pathway, might involve in anti-photoaging effects of the antioxidant peptides in HDF-a cells. Furthermore, an emulsion containing antioxidant peptides was prepared, and it was also found to inhibit the expression of MMPs and inflammation factors IL-6 and IL-1β in the mice exposed to UVB. Our results suggest that these antioxidant peptides might be applied as effective components in cosmetics for retarding skin photoaging in the future.

## 1 Introduction

Aging of skin is an inevitable biological process caused by both intrinsic and extrinsic factors, which not only influences the physiological functions of the skin, but also affects psychological wellbeing and social life (Sun J. et al., 2024; Mukherjee et al., 2011). Various extrinsic factors such as exposure to sunlight, air pollution, and nicotine, accelerate the skin aging process (Farage et al., 2008; Schikowski and Hüls, 2020). Exposure to ultraviolet B (UVB), in particular, plays the most important role in skin aging, also known as photoaging (Gu et al., 2020; Li et al., 2022).

UVB-induced skin photoaging primarily involves oxidative stress and inflammation (Shin et al., 2017; Wang et al., 2022). It has been demonstrated that excessive UVB irradiation can result in overproduction of the reactive oxygen species (ROS) in skin cells (Yang et al., 2019). ROS not only worsen the damage to skin cells, but also further enhance inflammatory response and facilitate the inflammatory cascade together with UVB, leading to overproduction of the cytokines such as IL-6, IL-1β etc., which in turn induces a production of more ROS (Hegedűs et al., 2020; Atalay et al., 2020). Moreover, ROS play a crucial role in regulating collagen metabolism and enhance expression of the matrix metalloproteinases (MMPs) in human skin (Patel et al., 2024; Zague et al., 2018). MMPs are a family of zinc-dependent endopeptidases (Coussens et al., 2002; Uria and Werb, 1998), among which, four members, interstitial collagenase (MMP1), gelatinase A (MMP2), stromelysin-1 (MMP3) and gelatinase B (MMP9), are especially responsible for the degradation of extracellular matrix (ECM) (Kammeyer and Luiten, 2015) and can fully degrade collagen together. In terms of the underlying molecular mechanisms, ROS are considered to be responsible for initiating activation of the mitogen-activated protein kinase (MAPK) signaling pathways through both direct and indirect ways (Boo, 2020; Fisher et al., 1998). The family of MAPKs comprises extracellular signal-regulated kinase (ERK), c-Jun N-terminal kinase (JNK), and p38 mitogen activated protein kinase (p38 MAPK), which are involved in regulating cell proliferation, differentiation, apoptosis, inflammation and collagen metabolism (Ferrer et al., 2002; Chang, 2012). In addition, UVB can be absorbed directly by DNA and RNA, thus activating DNA and RNA damage response pathways, which triggers multiple cascade effects, including the activation of MAPK pathways (Nk et al., 2024).

Researchers have found that some collagen hydrolysates and plant extracts exhibit significant anti-photoaging effects. For instances, Shin et al. reported that a combination of soy extract (SE) and *Haematococcus* extract (HE) can prevent UVB-induced photoaging through inhibiting mitogen-activated protein kinase (MAPK) phosphorylation and the transactivation of AP-1 which plays an important role in regulating MMP expression (Sun J. et al., 2024). Antioxidant peptides are a group of promising compounds thanks to their safety, bioavailability and strong capacities for ROS scavenging, DNA protection, and lipid peroxidation inhibition (Zu et al., 2024). Various peptides with strong antioxidant activities have been isolated from marine organisms, demonstrating remarkable preventive effects against UVB-induced damage both *in vitro* and *in vivo* (Doungapai et al., 2022; Han et al., 2022). Three peptides, GYTGL, LGATGL, and VLGL, which were isolated from gelatin hydrolysate of tilapia skin, effectively prevented UVB-induced photoaging in mouse embryonic fibroblasts through inhibiting MMP-1 activity and ROS production (Liping et al., 2018). Hu et al. reported that the peptide FTGML, derived from gelatin hydrolysate of grass carp scales, exhibited intracellular antioxidant activity and effectively inhibited p38 and JNK in the MAPK signaling pathways in B16F10 melanoma cells (Hu et al., 2022). In our previous study, we obtained three modified antioxidant peptides (WP5, LW5 and YY6) derived from marine organisms, which could effectively penetrate cell membrane and exhibited significantly enhanced antioxidant capacity against UVB-induced oxidative stress in human keratinocyte cells through reducing multiple oxidative factors as well as influencing the Keap1/Nrf2 antioxidant signal pathway (Huang et al., 2025).

In this study, we aim to investigate whether these antioxidant peptides exert protective effects on UVB-induced photoaging. Human epidermal fibroblast (HDF-a) cells irradiated with UVB were used to assess the anti-photoaging effects of the antioxidant peptides on cellular level, focusing on MMPs regulation and MAPK pathway modulation. Furthermore, we formulated an oil-in-water (O/W) emulsion containing the antioxidant peptides, and evaluated the anti-photoaging effects of the emulsion in mice irradiated with UVB.

## 2 Materials and methods

### 2.1 Materials and reagents

All cell culture media and supplements were obtained from Gibco (Gaithersburg, United States). Cell Counting Kit-8 (CCK-8) was purchased from DOJINDO China Co., Ltd. (Shanghai, China). HDF-a cells were purchased from Kefan Biotechnology Co., Ltd. (Guangzhou, China). Protein quantification and chemiluminescent detection was performed using the Bicinchoninic acid (BCA) assay kit and SuperSignal West Pico PLUS Chemiluminescent Substrate respectively, which were obtained from Thermo Fisher Scientific Inc. (Waltham, United States). ProLong Gold Antifade Reagent with DAPI (4′,6-diamidino-2-phenylindole), antibodies to β-actin, MMP2, MMP9, p38, phosphorylated p38 (p-p38), JNK, phosphorylated JNK (p-JNK) and alpha-1 type I collagen (COL1A1), and anti-rabbit IgG (Alexa Fluor® 594) were purchased from Cell Signaling Technology, Inc. (Boston, United States). Antibodies to MMP1 and MMP3 were acquired from Proteintech Group, Inc. (Wuhan, China). Senescence β-galactosidase staining kit (SA-β-Gal), lysis buffer for western and immunoprecipitation (IP), sodium dodecyl sulfate-polyacrylamide gel electrophoresis (SDS-PAGE) quick preparation kit, native gel sample loading buffer, coomassie blue staining kit and radioimmunoprecipitation assay (RIPA) buffer were purchased from Beyotime Biotech Co., Ltd. (Shanghai, China). Human MMP2 and MMP9 enzyme linked immunosorbent assay (ELISA) kits were obtained from UpingBio Co., Ltd. (Hubei, China). The antioxidant peptide sequences investigated in this article were are Trp-Pro-Trp-His-Trp (WPWHW, WP5) with a relative molecular mass of 810.92, Leu-Trp-His-Trp-His (LWHWH, LW5) with a relative molecular mass of 777.89, and Tyr-Tyr-Pro-Tyr-Trp-Leu (YYPYWL, YY6) with a relative molecular mass of 904.04. WPWHW, LWHWH, and YYPYWL, which were modified from WPDHW, LWHTH, and YYPYQL through a single amino acid substitution, respectively, which were derived from marine organisms, which were custom-synthesized by Chinapeptides Co., Ltd. (Wuhan, China) using solid-phase synthesis (SPPS) (purity >95%). All chemicals used in this study were of analytical grade or higher.

### 2.2 Establishment of UV-irradiated cell model

The UV-irradiated cell model was established as reported previously with slight modifications (Wang et al., 2022). Briefly, HDF-a cells were seeded in a 96-well plate at a density of 1.0 × 10^4^ cells/well (100 µL medium/well) and cultured for 24 h. After removing the culture medium, cells were washed three times with Dulbecco’s phosphate-buffered saline (DPBS) and covered with a thin DPBS layer for UVB irradiation. UVB exposure was performed using a TL20W/12 UVB Lamp (Philips, Germany) within the emission spectrum range of 280–320 nm (peak, 310–315 nm), and the intensity was monitored with a LS125-UVB-X0 UV radiometer (Shenzhen, China), and the cells were irradiated until cumulative dosages of 0, 5, 10, 15, 20, 30, 40 and 50 mJ/cm^2^ were reached, requiring 0, 25, 50, 75, 100, 150, 200, 250 s under the TL20W/12 UVB Lamp. After irradiation, HDF-a cells were washed 3 times with DPBS and cultured in new culture medium for 24 h. Cell viability was measured by CCK-8 assay, and the dosage of UVB that induced a decrease in HDF-a cell viability to approximately 50%–70% was chosen to establish the cell aging model as reported (Sun J.-M. et al., 2024).

### 2.3 Senescence-associated β-galactosidase staining (SA-β-Gal)

The expression of senescence-associated β-galactosidase (SA-β-Gal) in HDF-a cells was determined by SA-β-Gal staining kit according to the manufacturer’s instructions. In brief, HDF-a cells were fixed with β-Gal fixative for 15 min, washed three times with DPBS, incubated with the staining working solution, and maintained at 37 °C overnight. SA-β-Gal positive cells or areas were visualized using a fluorescence microscope (Leica K5 BZ03, Germany).

### 2.4 Viability assay of HDF-a cells after UV radiation (UVR)

The protective effects of antioxidant peptides against UVB-induced oxidative damage in HDF-a cells were evaluated according to a method previously reported with minor modifications (Uria and Werb, 1998). HDF-a cells were seeded in a 96-well (1.0 × 10^4^ cells/well) and a 24-well plate (3.0 × 10^4^ cells/well) and cultured overnight. After washing three times with DPBS, cells were covered with a thin DPBS layer and irradiated with UVB at dosages predetermined above. Immediately post-irradiation, the cells were treated with antioxidant peptides (24 h). Cell viability was assessed using a CCK-8 assay (vitamin C (100 μg/mL) as control). The cells in the 24-well plates were stained with SA-β-Gal staining kit as mentioned above. Images were acquired using fluorescence microscopy.

### 2.5 Gelatin zymography

Gelatin was used as a substrate for the proteolytic zymography assay as previously reported with minor modifications (Hwang et al., 2020). In brief, HDF-a cells were seeded in a 6-well plate at a density of 5.0 × 10^4^ cells/well and cultured overnight. After UVB irradiation, cells were treated with antioxidant peptides (24 h). Then, the cells were harvested and rinsed with cold phosphate buffer saline (PBS) twice, and lysed using lysis buffer for Western and IP containing 1% phenylmethanesulfonyl fluoride (PMSF). Following scraping and gentle pipetting to homogenize, lysates were incubated on ice for 15 min. After centrifugation (10,000 *g*, 15 min, 4 °C), supernatants were collected for immediate use or storage at −80 °C. Protein concentration was measured by BCA assay. The supernatant was prepared with native gel sample loading buffer, and subjected to SDS-PAGE with 10% acrylamide gels. After the electrophoresis, the gel was washed for 30 min twice with 2.5% Triton X-100 and incubated in zymography reaction buffer at 37 °C for an additional 24 h. The gel was then stained with Coomassie blue R-250 for 15 min, destained and imaged using an iBright gel imaging system (FL1500, United States).

### 2.6 Determination of protein expression

Western blotting was performed to detect the protein expression of β-actin, MMP1, MMP2, MMP3, MMP9, JNK, p-JNK, p38, p-p38, IL-6, and IL-1β in HDF-a cells and mouse skin (β-actin, MMP2, MMP9, p38, phosphorylated p38 (p-p38), JNK, phosphorylated JNK (p-JNK) and alpha-1 type I collagen (COL1A1) were purchased from Cell Signaling Technology, Inc. (Boston, United States); MMP1 and MMP3 were acquired from Proteintech Group, Inc. (Wuhan, China)). Cell culture, treatment, and protein extraction were similar as described in gelatin zymography. The mouse dorsal skin tissues were lysed with RIPA containing 1% PSMF and 1% phosphatase inhibitor complex (100×) using a tissue homogenate machine (SKGZ, China). The supernatant was mixed with protein loading buffer, then boiled for 7 min and subjected to SDS-PAGE using 10% acrylamide gels. The proteins were transferred onto a polyvinylidene difluoride (PVDF) membrane, which was then blocked with 5% non-fat milk or 5% bovine albumin (BSA) at room temperature for 2 h, and incubated with primary antibodies overnight (4 °C), then incubated with horseradish peroxidase (HRP) conjugated secondary antibodies (1 h, room temperature). The intensity of the specific immunoreactive bands was detected using an enhanced chemiluminescence apparatus and then quantified with ImageJ software (Schneider et al., 2012).

### 2.7 Cytokine quantification by enzyme linked immunosorbent assay (ELISA)

Following identical culture and treatment as described for gelatin zymography, the HDF-a cells culture supernatants were collected for quantification of MMP2 and MMP9 by ELISA kits.

### 2.8 Immunofluorescent staining

Immunofluorescent staining was performed as described previously with some modifications (Gu et al., 2022). Briefly, HDF-a cells were seeded into 12-well plates (4.0 × 10^4^ cells/well), following identical culture and treatment as described for gelatin zymography, the cells were fixed with 4% paraformaldehyde for 15 min at room temperature and then permeabilized with 0.5% Triton X-100 solution for 10 min. After washing with PBS three times, the cells were blocked with PBS containing 3% goat serum and 1% BSA for 1 h, and then incubated overnight at 4 °C with primary antibody against COL1A1. After washing three times with PBS, the cells were incubated with Alexa Fluor 594-conjugated Goat Anti-Rabbit IgG (H+L) secondary antibody. Nuclei were counterstained using ProLong Gold Antifade Reagent with DAPI. Images were acquired using a confocal microscope (Leica K5 BZ03, Germany).

### 2.9 Mouse model of skin photoaging

All animal experiments were performed according to the Guide for the Care and Use of Laboratory Animals and were approved by the Medical Ethics Committee of the Naval Medical University (IACUC protocol number NMU-2021Q020). All mice (Institute of Cancer Research mice) were kept at room temperature (24 °C ± 1 °C) and under a 12 h light/dark cycle, and were freely provided with water and standard diet. After 1 week of acclimation, an area of mouse dorsal skin was shaved, and the mice were randomly divided into six groups: negative control, UVB + PBS, UVB + emulsion substrate (the detail process for the fabrication and complete formulation of the emulsion can be found in Supplementary Table S1; Supplementary Figure S1), UVB + emulsion containing 1% antioxidant peptide (WP5, LW5 or YY6). All mice, except those from the negative control group, were topically applied with PBS or emulsion on their bare dorsal skin in advance, and then were irradiated to a cumulative UVB dosage of 20 mJ/cm^2^ with a UVB Lamp. Twenty-four hours after irradiation, all the mice were anesthetized and killed by cervical dislocation. Dorsal skin samples were collected and then were either fixed in 4% paraformaldehyde and 2.5% glutaraldehyde solution or snap-frozen in liquid nitrogen and stored at −80 °C until further use.

### 2.10 Histological examination

The dorsal skin tissues were removed and washed with cold saline, and then fixed in a 4% paraformaldehyde solution overnight. After dehydration through a graded ethanol series, the tissues were embedded in paraffin and sectioned. Hematoxylin-eosin (H&E) staining and Masson’s trichrome staining (Servicebio, Wuhan, China) were used to assess histopathological changes and collagen deposition, respectively. Epidermal and dermal thicknesses were measured using CaseView software.

### 2.11 Statistical analysis

Statistical analyses were performed using GraphPad Prism 9. Between-group differences were assessed by unpaired *t*-test (two groups) or one-way ANOVA (≥3 groups) with *p*-value <0.05 was considered statistically significant.

## 3 Results

### 3.1 Protective effects of the antioxidant peptides on UVB-irradiated HDF-a cells

**FIGURE 1:**
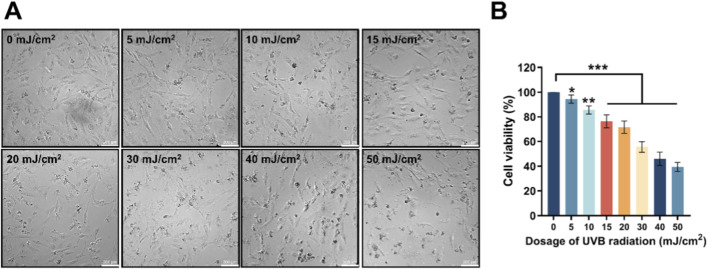
The influence of different dosages of UVB radiation on the morphology and viability of HDF-a cells. **(A)** The sequential morphological changes of HDF-a cells after 24 h by various dosages of UVB radiation. **(B)** Effect of different UVB radiation dosages on the viability of HDF-a cells. *P < 0.05, **P < 0.01, ***P < 0.001 vs. control group (0 mJ/cm^2^). Bar = 200 μm.

HDF-a cells were irradiated with different dosages of UVB (0, 5, 10, 15, 20, 30, 40 and 50 mJ/cm^2^). Progressive dose-dependent morphological changes were observed: cell numbers declined sequentially with increasing UVB intensity, accompanied by cell rounding and nuclear condensation, indicating substantial cell damage (Figure 1A). Correspondingly, the cell viability decreased as the dosage of UVB increased from 0 to 50 mJ/cm^2^ (Figure 1B). Therefore, 30 mJ/cm^2^, which induced a reduction of 30%–50% in HDF-a cell viability, was selected as the optimal radiation dosage for establishing a UVB-irradiated *in vitro* photoaging model according to the reports (Han et al., 2022; Sun J.-M. et al., 2024).

To determine the optimal concentrations of WP5, LW5 and YY6 for their protective effects against UVB-induced damage, HDF-a cells were post-treated with each of the peptides at graded concentrations (0.063, 0.125, 0.25, 0.5 mg/mL) following UVB irradiation. Cell viability analysis revealed significantly higher survival rates in the treatment groups with 0.25 mg/mL antioxidant peptides compared to the radiation model group. Meanwhile, no statistically significant difference was observed between 0.25 mg/mL and 0.5 mg/mL groups (Supplementary Figure S2). Therefore, 0.25 mg/mL was selected as the optimal concentration of WP5, LW5, and YY6 for subsequent experiments.

**FIGURE 2:**
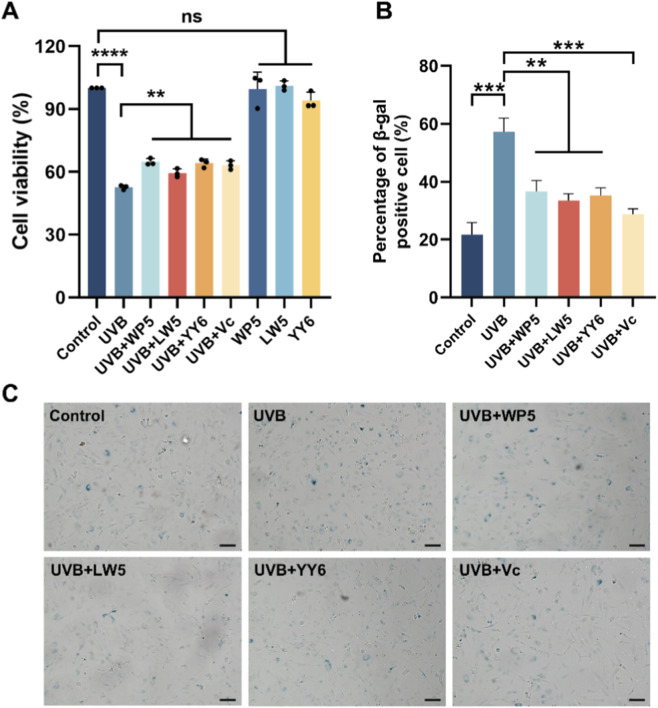
Protective effects of the three antioxidant peptides against UVB radiation in HDF-a cells. **(A)** The antioxidant peptides (0.25 mg/mL) recovered the viability of HDF-a cells irradiated by UVB. Cell viabilities were determined using CCK-8 assay kit. **(B)** The antioxidant peptides reduced the proportion of β-gal-positive cells induced by UVB radiation. **(C)** Comparison of the representative images of SA-β-gal-positive cells in different treatment groups. Bar = 100 μm. (ns, no significance, **P < 0.01, ***P < 0.001, n = 3).

As shown in Figure 2A, the viability of HDF-a cells decreased after UVB irradiation, but then significantly recovered through incubation with WP5, LW5, YY6 and vitamin C. In addition, Our data suggested that SA-β-gal-positive cells increased markedly following the radiation of UVB. However, the number of SA-β-gal-positive cells decreased significantly after treatment by antioxidant peptide compared to the UVB radiation model group (Figures 2B,C). These findings collectively indicated that WP5, LW5, YY6 and vitamin C effectively alleviated UVB-induced photoaging in human dermal cells.

### 3.2 Antioxidant peptides alleviated photoaging through MAPK signaling pathway in HDF-a cells exposed to UVB radiation

**FIGURE 3:**
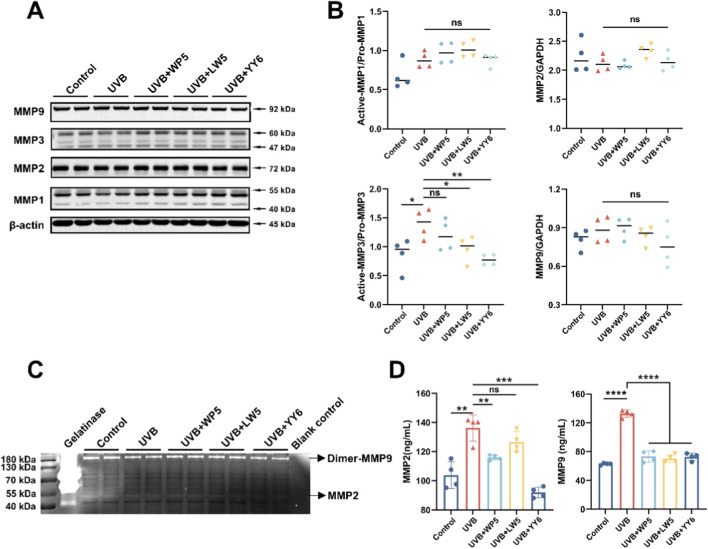
The antioxidant peptides protected HDF-a cells from UVB-induced aging by inhibiting the expression of MMPs. **(A)** Western blotting of MMP1, MMP2, MMP3, MMP9 in HDF-a cell lysates from different treatment groups. Quantitation is shown on the right **(B)**. **(C)** The enzymatic activity of MMP2 and MMP9 in HDF-a cell lysates evaluated by gelatin zymography. **(D)** The protein levels of MMP2 and MMP9 in HDF-a cell supernatants measured by ELISA. (ns, no significance, *P < 0.05, **P < 0.01, ***P < 0.001, n = 4).

To further explore the potential molecular mechanism underlying UVB-induced dermal cell senescence and collagen reduction, HDF-a cells were irradiated with UVB (30 mJ/cm^2^) *in vitro*. The results of Western blotting and gelatin zymography confirmed that the protein level of active MMP3 in cell lysates was evidently elevated in the UVB radiation model group compared to normal control group (Figures 3A,B), while was significantly dropped after treatment with the antioxidant peptides LW5 and YY6. However, neither the protein expression nor enzymatic activity of MMP2 and MMP9 in cell lysates showed significant alterations after UVB exposure (Figures 3A–C), suggesting a possible extracellular translocation of MMP2 and MMP9. Subsequent ELISA assay revealed a significant elevation of MMP2 and MMP9 levels in the culture supernatants from the UVB radiation model group, indicating that UVB might drive the secretory upregulation of MMP2 and MMP9. As expected, the protein levels of MMP2 and MMP9 in culture supernatants significantly decreased after antioxidant peptide treatment (Figure 3D). These findings suggested that the three antioxidant peptides could protect HDF-a cells from UVB-induced aging by inhibiting the expression of MMPs.

**FIGURE 4:**
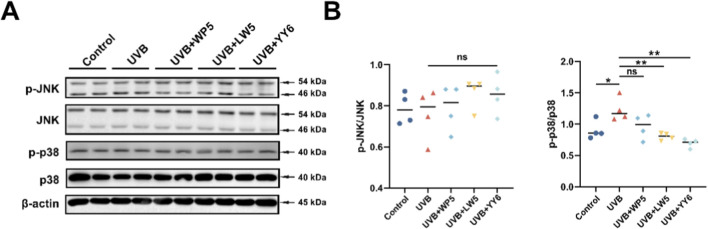
The antioxidant peptides protected HDF-a cells from UVB-induced aging through suppressing MAPK signaling. **(A)** Western blotting of p38, phospho-p38, JNK and phospho-JNK in HDF-a cell lysates from different treatment groups. Quantitation is shown on the right **(B)**. (ns, no significance, *P < 0.05, **P < 0.01, ***P < 0.001, n = 4).

In addition, UVB exposure markedly enhanced p38 phosphorylation compared to normal control group (Figures 4A,B), while the antioxidant peptides LW5 and YY6 significantly alleviated the increase in p38 phosphorylation caused by UVB irradiation. Intriguingly, we failed to observe an increase in JNK phosphorylation, which belongs to another pathway of MAPK and is also known to upregulate MMPs. These results indicated that the antioxidant peptides might protect HDF-a cells from UV-induced photoaging through suppressing p38 MAPK pathway.

### 3.3 Antioxidant peptides suppressed type I collagen degradation in HDF-a cells exposed to UVB radiation

**FIGURE 5:**
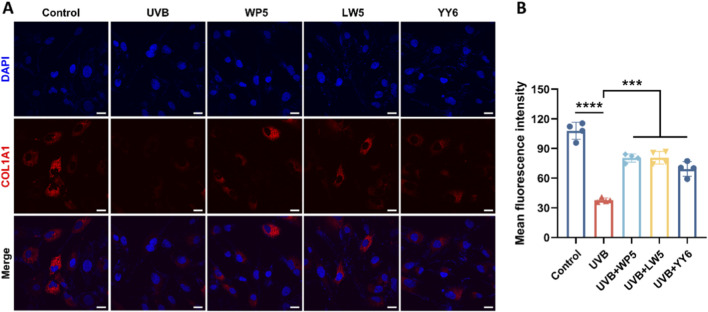
The antioxidant peptides suppressed UVB-induced depletion of collagen type I precursor (COL1A1). **(A)** Representative immunofluorescence images of COL1A1 photographed by confocal microscopy. COL1A1 was stained with Alexa Fluor 594-conjugated antibody (red), and nuclei were stained with DAPI (blue). Scale bar = 20 μm. Quantitation is shown on the right **(B)**. (***P < 0.001, ****P < 0.0001, n = 4).

Protein expression of type I collagen precursor (COL1A1) in HDF-a cells was assessed by immunofluorescence assay. As illustrated in Figure 5, COL1A1 expression decreased evidently in the UVB radiation model group compared to the normal control group. In contrast, COL1A1 expression recovered significantly after treatment with antioxidant peptides. These data conclusively demonstrated that the antioxidant peptides counteracted UVB-mediated collagen depletion in HDF-a cells, thereby ameliorating photoaging.

### 3.4 Emulsion containing antioxidant peptides retarded UVB-induced epidermal thickening

**FIGURE 6:**
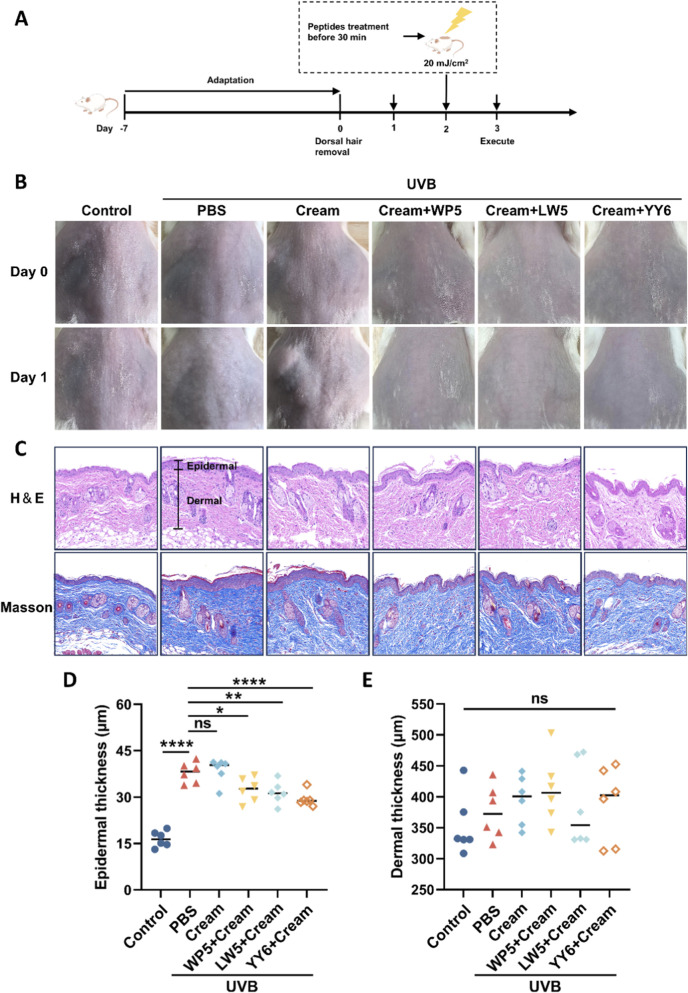
Emulsion containing antioxidant peptides protected the dorsal skin of mice against UVB-induced damage. **(A)** The experimental procedure for evaluating the protective effects against UVB-induced skin acute injury in mice. **(B)** Gross observation of the dorsal skin of mice with different treatment protocols. **(C)** Epidermal thickness was observed by H&E staining. The black line indicates the epidermal layer of the skin tissue in each group. Scale bar = 100 μm. **(D)** Statistical analysis of epidermal thickness of the mice with different treatment protocols. **(E)** Statistical analysis of dermal thickness of the mice with different treatment protocols. (ns, no significance, *P < 0.05, **P < 0.01, ****P < 0.001, n = 6).

UVB radiation has a direct action on the cutaneous epidermis due to its weaker penetrating property compared to UVA (Yang et al., 2019). Therefore, we evaluated the impact of UVB radiation on the epidermal layer and the protective effects of the antioxidant peptides in mouse model of skin photoaging. Histological examination by hematoxylin and eosin (H&E) staining (Figure 6) revealed thickened epidermal, but not dermal, layers in the mice treated with PBS or emulsion substrate in advance and then exposed to UVB, compared to the mice in normal control group. Meanwhile, the mice treated with the emulsion containing antioxidant peptides demonstrated a reduction in epidermal thickness compared to those in PBS and cream substrate groups. The data indicated that the emulsion containing antioxidant peptides effectively protected the dorsal skin of mice against UVB-induced damage.

### 3.5 Emulsion containing antioxidant peptides alleviated photoaging and suppressed inflammation response in mice irradiated with UVB

**FIGURE 7:**
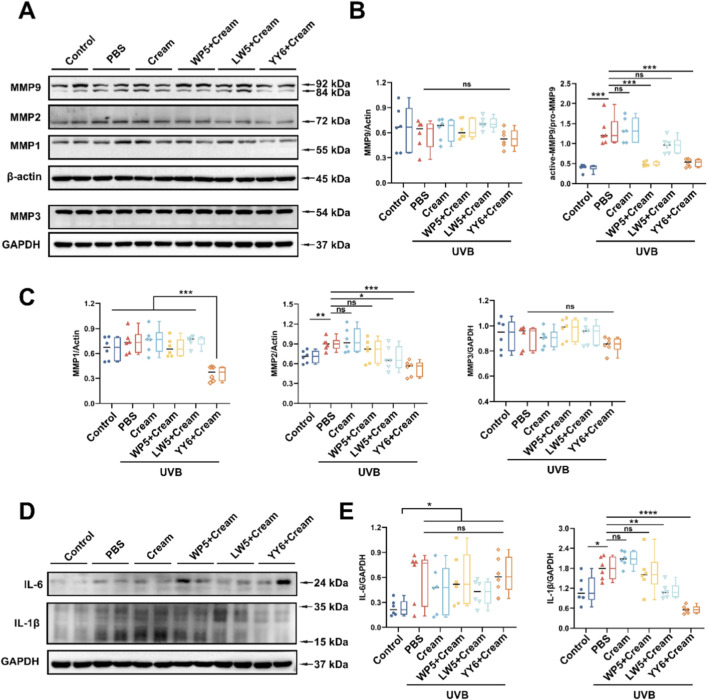
Suppressing effects of the emulsion containing antioxidant peptides on UVB-induced MMPs expression and inflammatory factors. **(A)** Western blotting of MMP1, MMP2, MMP3 and MMP9 in the dorsal skin tissues of mice with different treatment protocols. **(B)** Quantitation of the MMP9 expression in **(A)**. **(C)** Quantitation of the MMP1, MMP2 and MMP3 expression in **(A)**. **(D)** Western blotting of IL-6 and IL-1β in the dorsal skin tissues of mice with different treatment protocols. Quantitation is shown on the right **(E)**. (ns, no significance, *P < 0.05, **P < 0.01, ****P < 0.001, n = 6).

MMPs and inflammatory factors have been reported to play crucial roles in cutaneous photoaging. Thus, we examined the expression of MMPs (MMP1, MMP2, MMP3 and MMP9) and pro-inflammatory cytokines (IL-6 and IL-1β) in mouse model of skin photoaging. As shown in Figure 7, there was a marked increase in protein expression of MMP2, active MMP9, IL-6 and IL-1β in the mice treated with PBS or emulsion substrate in advance and then exposed to UVB compared to the mice in normal control group, but we failed to observe the synchronous increase of MMP1 and MMP3. Notably, the emulsion containing WP5 specifically attenuated the activation of MMP9 but without modulating IL-6 and IL-1β (Figures 7A,B,D,E). As for the emulsion containing LW5, a dual effect was demonstrated, which suppressed both MMP-9 activation and MMP-2 expression, and concurrently downregulated IL-1β level (Figure 7). The efficacy of the emulsion containing YY6 was comparable to that of the emulsion containing LW5, simultaneously exerting anti-inflammatory and anti-photoaging effects.

## 4 Discussion

UVR is one of the most harmful environmental factors driving cutaneous photoaging (Gaweł-Bębe et al., 2020). Prolonged exposure to UVR induces multifaceted cellular damage, including DNA and RNA mutations, impaired protein synthesis, and metabolic disorders, and the histopathological alterations predominantly manifest in the dermal ECM, which is a process mechanistically linked to MMPs overexpression (Nk et al., 2024; K et al., 2021). In fact, there is literature indicating that UVB irradiation can induce the expression of MMPs, which in turn degrades the ECM, leading to photoaging (Vayalil et al., 2004). Therefore, the inhibition of UVB-induced MMPs overexpression may be a promising strategy to prevent photoaging. In the present study, we unveiled the anti-photoaging potential of the three antioxidant peptides WP5, LW5 and YY6 using models of UVB radiation-induced dermal fibroblast senescence and skin photoaging. We found that premature senescence was induced by UVB irradiation in HDF-a cells using SA-β-Gal staining. Afterwards, incubation with antioxidant peptides or vitamin C effectively alleviated cell senescence, exerting anti-photoaging effects. Molecular profiling of HDF-a cells revealed the activation and upregulation of MMP2, MMP3 and MMP9 after UVB irradiation. However, the antioxidant peptides LW5 and YY6 significantly suppressed the activation and expression of MMP2, MMP3 and MMP9 in HDF-a cells, while WP5 inhibited MMP2 and MMP9 activation but without affecting MMP3. This differential efficacy suggests that these peptides may engage distinct signaling pathways or molecular targets to counteract UVB damage. Notably, the peptides’ performance, compared to the positive control vitamin C, hints at potential advantages in terms of stability or specificity worthy of further investigation and YY6 exhibited an inhibitory effect on a wider range of MMPs. These compelling *in vitro* results highlight the promise of LW5, YY6, and WP5 as candidate agents for developing innovative therapeutic or cosmetic strategies against skin photoaging.

Previous research has suggested the pivotal role of MAPK signaling pathways in photoaging, which are closely related to the expression of MMPs (Rittié and Fisher, 2002; Fernando et al., 2020). In the present study, UVB irradiation elevated the expression of p-p38/p38, but did not affect p-JNK/JNK. Nevertheless, LW5 and YY6 alleviated the increase of p-p38/p38 expression caused by UVB irradiation, which might be related to their inhibition on the activation and expression of MMP2, MMP3 and MMP9 in HDF-a cells. In addition, the suppression on the expression of alpha-1 type I collagen (COL1A1) that is the precursor of type I collagen may lead to the degradation of type I collagen (Talwar et al., 1995). Therefore, we also detected the expression of COL1A1 in HDF-a cells by immunofluorescence. As expected, the COL1A1 was evidently depleted after UVB exposure in HDF-a cells, however, the antioxidant peptides obviously recovered the expression of COL1A1. These findings indicated that the three antioxidant peptides can alleviate the degradation of extracellular matrix in UVB induced photoaging.

To further investigate the preventative effects of antioxidant peptides on UVB-induced photoaging *in vivo*, an O/W emulsion containing the antioxidant peptides was prepared. UVB irradiation caused acute dermal damage characterized by diffuse edema and thickening in the dorsal skin of mice, and topical application of emulsion containing antioxidant peptides markedly retarded the edema and thickening of skin, indicating that the antioxidant peptides could protect the bare skin against UVB-induced damage. Further investigation on the molecular mechanism showed that UVB also upregulated the expression of MMP2 and MMP9 that was consistent with cellular experiments, which are the key proteins causing skin photoaging, and raised the levels of inflammatory factors IL-6 and IL-1β, which was consistent with previous studies (Xie et al., 2021; Song et al., 2023). However, we observed that while UVB irradiation enhanced MMP3 activation in cellular experiments, a corresponding increase in MMP3 expression or activation was not detected in the mouse model. This discrepancy could be related to various factors such as the irradiation dose, interspecies differences, and the distinct physicochemical environments between *in vitro* and *in vivo* settings. It is noteworthy that YY6 not only effectively inhibited the activation of MMP3 and the secretion levels of MMP2 and MMP9 in cellular experiments, but also demonstrated a consistent ability to suppress the expression or activation of MMP2 and MMP9 in mouse experiments, along with a certain anti-inflammatory efficacy. Taken together, the emulsion containing antioxidant peptides effectively suppressed the expression of MMP2 or MMP9 and reduced the production of inflammatory factors IL-6 and IL-1β, albeit their effects were slightly different. These results demonstrated that the three antioxidant peptides possessed a potential antiphotoaging effect through downregulating MMPs and inflammation factors.

## 5 Conclusion

In summary, the three antioxidant peptides WP5, LW5 and YY6 exhibited a marked protective effect against UVB-induced photoaging in HDF-a cells, and the underlying mechanism might be associated with the downregulation of MMPs expression and suppression of collagen I degradation through the modulation of p38 MAPK signaling pathways. Furthermore, an O/W emulsion containing the antioxidant peptides demonstrated an outstanding anti-photoaging efficacy in the mice exposed to UVB by suppressing the expression of MMPs, IL-6, and IL-1β. Our research provided new perspectives on the future application of antioxidant peptides as natural anti-aging ingredients in cosmetic products. However, this study only demonstrated the anti-photoaging and anti-inflammatory effects of the modified peptide cream in the acute injury responses, further investigation is required in a UVB irradiation-induced chronic skin injury model.

## Data Availability

The original contributions presented in the study are included in the article/Supplementary Material, further inquiries can be directed to the corresponding authors.

## References

[B1] Atalay S. Dobrzyńska I. Gęgotek A. Skrzydlewska E. (2020). Cannabidiol protects keratinocyte cell membranes following exposure to UVB and hydrogen peroxide. Redox Biol. 36, 101613. 10.1016/j.redox.2020.101613 32863232 PMC7327251

[B2] Boo Y. C. (2020). Emerging strategies to protect the skin from ultraviolet rays using plant-derived materials. Antioxid. Basel Switz. 9, 637. 10.3390/antiox9070637 32708455 PMC7402153

[B3] Chang A. Y. (2012). Pro-life role for c-Jun N-terminal kinase and p38 mitogen-activated protein kinase at rostral ventrolateral medulla in experimental brain stem death. J. Biomed. Sci. 19, 96. 10.1186/1423-0127-19-96 23157661 PMC3533910

[B4] Coussens L. M. Fingleton B. Matrisian L. M. (2002). Matrix metalloproteinase inhibitors and cancer: trials and tribulations. Science 295, 2387–2392. 10.1126/science.1067100 11923519

[B5] Doungapai C. Siriwoharn T. Malila Y. Autsavapromporn N. Makkhun S. Yarnpakdee S. (2022). UV-B protective and antioxidant activities of protein hydrolysate from sea cucumber (*Holothuria scabra*) using enzymatic hydrolysis. Front. Mar. Sci. 9, 892255. 10.3389/fmars.2022.892255

[B6] Farage M. A. Miller K. W. Elsner P. Maibach H. I. (2008). Intrinsic and extrinsic factors in skin ageing: a review. Int. J. Cosmet. Sci. 30, 87–95. 10.1111/j.1468-2494.2007.00415.x 18377617

[B7] Fernando I. P. S. Dias M. K. H. M. Madusanka D. M. D. Han E. J. Kim M. J. Jeon Y.-J. (2020). Fucoidan refined by Sargassum confusum indicate protective effects suppressing photo-oxidative stress and skin barrier perturbation in UVB-induced human keratinocytes. Int. J. Biol. Macromol. 164, 149–161. 10.1016/j.ijbiomac.2020.07.136 32682044

[B8] Ferrer I. Blanco R. Carmona M. Puig B. Domínguez I. Viñals F. (2002). Active, phosphorylation-dependent MAP kinases, MAPK/ERK, SAPK/JNK and p38, and specific transcription factor substrates are differentially expressed following systemic administration of kainic acid to the adult rat. Acta Neuropathol. (Berl.) 103, 391–407. 10.1007/s00401-001-0481-9 11904760

[B9] Fisher G. J. Talwar H. S. Lin J. Lin P. McPhillips F. Wang Z. (1998). Retinoic acid inhibits induction of c-Jun protein by ultraviolet radiation that occurs subsequent to activation of mitogen-activated protein kinase pathways in human skin *in vivo* . J. Clin. Invest. 101, 1432–1440. 10.1172/JCI2153 9502786 PMC508699

[B10] Gaweł-Bęben K. Kukula-Koch W. Hoian U. Czop M. Strzępek-Gomółka M. Antosiewicz B. (2020). Characterization of Cistus × incanus L. and Cistus ladanifer L. extracts as potential multifunctional antioxidant ingredients for skin protecting cosmetics. Antioxidants 9, 202. 10.3390/antiox9030202 32121584 PMC7139296

[B11] Gu Y. Han J. Jiang C. Zhang Y. (2020). Biomarkers, oxidative stress and autophagy in skin aging. Ageing Res. Rev. 59, 101036. 10.1016/j.arr.2020.101036 32105850

[B12] Gu Y. Xue F. Xiao H. Chen L. Zhang Y. (2022). Bamboo leaf flavonoids suppress oxidative stress-induced senescence of HaCaT cells and UVB-induced photoaging of mice through p38 MAPK and autophagy signaling. Nutrients 14, 793. 10.3390/nu14040793 35215447 PMC8876272

[B13] Han S. H. Ballinger E. Choung S.-Y. Kwon J. Y. (2022). Anti-photoaging effect of hydrolysates from pacific whiting skin via MAPK/AP-1, NF-κB, TGF-β/Smad, and Nrf-2/HO-1 signaling pathway in UVB-induced human dermal fibroblasts. Mar. Drugs 20, 308. 10.3390/md20050308 35621960 PMC9147990

[B14] Hegedűs C. Juhász T. Fidrus E. Janka E. A. Juhász G. Boros G. (2020). Cyclobutane pyrimidine dimers from UVB exposure induce a hypermetabolic state in keratinocytes via mitochondrial oxidative stress. Redox Biol. 38, 101808. 10.1016/j.redox.2020.101808 33264701 PMC7708942

[B15] Hu Z. Sha X. Zhang L. Huang S. Tu Z. (2022). Effect of grass carp scale collagen peptide FTGML on cAMP-PI3K/Akt and MAPK signaling pathways in B16F10 melanoma cells and correlation between anti-melanin and antioxidant properties. Foods Basel Switz. 11, 391. 10.3390/foods11030391 35159541 PMC8834497

[B16] Huang Y. He Q. Zhang P. Song J. Wang Y. Zhu S. (2025). Single amino acid substitution analogs of marine antioxidant peptides with membrane permeability exert a marked protective effect against ultraviolet-B induced damage. J. Photochem. Photobiol. B 264, 113120. 10.1016/j.jphotobiol.2025.113120 39922038

[B17] Hwang D. H. Lee H. Choudhary I. Kang C. Chae J. Kim E. (2020). Protective effect of epigallocatechin-3-gallate (EGCG) on toxic metalloproteinases-mediated skin damage induced by Scyphozoan jellyfish envenomation. Sci. Rep. 10, 18644. 10.1038/s41598-020-75269-1 33122740 PMC7596074

[B18] K V. O K. A M. Sn S. Aa A. Va T. (2021). Keratinocyte death by ferroptosis initiates skin inflammation after UVB exposure. Redox Biol. 47, 102143. 10.1016/j.redox.2021.102143 34592565 PMC8487085

[B19] Kammeyer A. Luiten R. M. (2015). Oxidation events and skin aging. Ageing Res. Rev. 21, 16–29. 10.1016/j.arr.2015.01.001 25653189

[B20] Li M. Lyu X. Liao J. Werth V. P. Liu M.-L. (2022). Rho kinase regulates neutrophil NET formation that is involved in UVB-induced skin inflammation. Theranostics 12, 2133–2149. 10.7150/thno.66457 35265203 PMC8899566

[B21] Liping S. Qiuming L. Jian F. Xiao L. Yongliang Z. (2018). Purification and characterization of peptides inhibiting MMP-1 activity with c terminate of Gly-Leu from simulated gastrointestinal digestion hydrolysates of tilapia (*Oreochromis niloticus*) skin gelatin. J. Agric. Food Chem. 66, 593–601. 10.1021/acs.jafc.7b04196 29272917

[B22] Mukherjee P. K. Maity N. Nema N. K. Sarkar B. K. (2011). Bioactive compounds from natural resources against skin aging. Phytomedicine 19, 64–73. 10.1016/j.phymed.2011.10.003 22115797

[B23] Nk, M. C S. Zy Y. Jj, N. Kh L. Tm Y.-B. Jl J. Em H. (2024). The ribotoxic stress response drives UV-mediated cell death. Cell. 10.1016/j.cell.2024.05.018 PMC11246228 38843833

[B24] Patel S. K. Vikram A. Pathania D. Chugh R. Gaur P. Prajapati G. (2024). Allergic potential and molecular mechanism of skin sensitization of cinnamaldehyde under environmental UVB exposure. Chemosphere 368, 143508. 10.1016/j.chemosphere.2024.143508 39384131

[B25] Rittié L. Fisher G. J. (2002). UV-light-induced signal cascades and skin aging. Ageing Res. Rev. 1, 705–720. 10.1016/s1568-1637(02)00024-7 12208239

[B26] Schikowski T. Hüls A. (2020). Air pollution and skin aging. Curr. Environ. Health Rep. 7, 58–64. 10.1007/s40572-020-00262-9 31927691

[B27] Schneider C. A. Rasband W. S. Eliceiri K. W. (2012). NIH image to ImageJ: 25 years of image analysis. Nat. Methods 9, 671–675. 10.1038/nmeth.2089 22930834 PMC5554542

[B28] Shin J. Kim J.-E. Pak K.-J. Kang J. I. Kim T.-S. Lee S.-Y. (2017). A combination of soybean and haematococcus extract alleviates ultraviolet b-induced photoaging. Int. J. Mol. Sci. 18, 682. 10.3390/ijms18030682 28327532 PMC5372692

[B29] Song B. Liu D. Liu T. C. Li K. Wang S. Liu J. (2023). The combined effect of commercial tilapia collagen peptides and antioxidants against UV-induced skin photoaging in mice. Food Funct. 14, 5936–5948. 10.1039/D3FO01516E 37337869

[B30] Sun J. Xie X. Song Y. Sun T. Liu X. Yuan H. (2024a). Selenomethionine in gelatin methacryloyl hydrogels: modulating ferroptosis to attenuate skin aging. Bioact. Mater. 35, 495–516. 10.1016/j.bioactmat.2024.02.013 38404642 PMC10885793

[B31] Sun J.-M. Liu Y.-X. Liu Y.-D. Ho C.-K. Tsai Y.-T. Wen D.-S. (2024b). Salvianolic acid B protects against UVB-Induced skin aging via activation of NRF2. Phytomedicine Int. J. Phytother. Phytopharm. 130, 155676. 10.1016/j.phymed.2024.155676 38820663

[B32] Talwar H. S. Griffiths C. E. Fisher G. J. Hamilton T. A. Voorhees J. J. (1995). Reduced type I and type III procollagens in photodamaged adult human skin. J. Invest. Dermatol. 105, 285–290. 10.1111/1523-1747.ep12318471 7543550

[B33] Uria J. A. Werb Z. (1998). Matrix metalloproteinases and their expression in mammary gland. Cell Res. 8, 187–194. 10.1038/cr.1998.19 9791732

[B34] Vayalil P. K. Mittal A. Hara Y. Elmets C. A. Katiyar S. K. (2004). Green tea polyphenols prevent ultraviolet light-induced oxidative damage and matrix metalloproteinases expression in mouse skin. J. Invest. Dermatol. 122, 1480–1487. 10.1111/j.0022-202X.2004.22622.x 15175040

[B35] Wang Z.-Y. Li A. Huang X. Bai G.-L. Jiang Y.-X. Li R.-L. (2022). HSP27 protects skin from ultraviolet b -induced photodamage by regulating autophagy and reactive oxygen species production. Front. Cell Dev. Biol. 10, 852244. 10.3389/fcell.2022.852244 35445017 PMC9014213

[B36] Xie C. Fan Y. Yin S. Li Y. Liu N. Liu Y. (2021). Novel amphibian-derived antioxidant peptide protects skin against ultraviolet irradiation damage. J. Photochem. Photobiol. B 224, 112327. 10.1016/j.jphotobiol.2021.112327 34628205

[B37] Yang Y. Wu R. Sargsyan D. Yin R. Kuo H.-C. Yang I. (2019). UVB drives different stages of epigenome alterations during progression of skin cancer. Cancer Lett. 449, 20–30. 10.1016/j.canlet.2019.02.010 30771437 PMC6411449

[B38] Zague V. do Amaral J. B. Rezende Teixeira P. de Oliveira Niero E. L. Lauand C. Machado-Santelli G. M. (2018). Collagen peptides modulate the metabolism of extracellular matrix by human dermal fibroblasts derived from sun-protected and sun-exposed body sites. Cell Biol. Int. 42, 95–104. 10.1002/cbin.10872 28906033

[B39] Zu X. Zhao Q. Liu W. Guo L. Liao T. Cai J. (2024). Sturgeon (*Acipenser schrenckii*) spinal cord peptides: antioxidative and acetylcholinesterase inhibitory efficacy and mechanisms. Food Chem. 461, 140834. 10.1016/j.foodchem.2024.140834 39153375

