# Supplementary material for: Antiphotoaging effects of a group of antioxidant peptides through downregulating matrix metalloproteinases and inflammation factors
Source: Front Cell Dev Biol. 2025 Nov 27;13:1649391. doi: 10.3389/fcell.2025.1649391 (PMC12695852; doi:10.3389/fcell.2025.1649391)

**Supplementary Information**

**1. Materials**

**Table S1.** Formulas of the emulsion.

| **Reagents** | **Manufacturer** | **Ratio** |
| --- | --- | --- |
| Stearic acid | Aladdin | 6% |
| Span 80 | Maya Reagent | 1.6% |
| 1-Octadecanol | Aladdin | 6% |
| Paraffin liquid | Aladdin | 9% |
| Vaseline | Aladdin | 6% |
| Tween 80 | Macklin | 4.4% |
| Glycerol | Aladdin | 10% |
| Sorbic acid | Aladdin | 0.2% |
| UP water | Millipore up water system | 55.8% |
| Antioxidant peptide | Synthesized by Chinapeptides Co., Ltd. | 1% |

**2. Methods**

Preparation of oil phase: stearic acid, Span 80, 1-octadecanol, paraffin liquid, and vaseline were accurately weighed according to the ratio in Table S1. The mixture was stirred under a heating temperature of 80°C with a stirring speed of 400 rpm. Preparation of water phase: Tween 80, glycerol, and sorbic acid were accurately weighed. Sorbic acid was first dissolved in ultrapure (UP) water, and then Tween 80 and glycerol were added successively. The mixture was stirred under a heating temperature of 80°C with a stirring speed of 400 rpm. Subsequently, the water phase was gradually added to the oil phase, and the combined mixture was stirred under a heating temperature of 80°C with a stirring speed of 400 rpm for 20 min. After that, the whole emulsion was gradually cooled down to 25°C, an appropriate volume of emulsion was collected, and then one of the antioxidant peptides (WP5, LW5 and YY6) was incorporated to obtain the final lotion.

**Figure S1.** Microscopic images of the lotion containing antioxidant peptide. (A) Unstained. (B) Methylene blue staining. (Scale bar = 200 μm). (C) Unstained. (D) Methylene blue staining. (Scale bar = 50 μm)

**
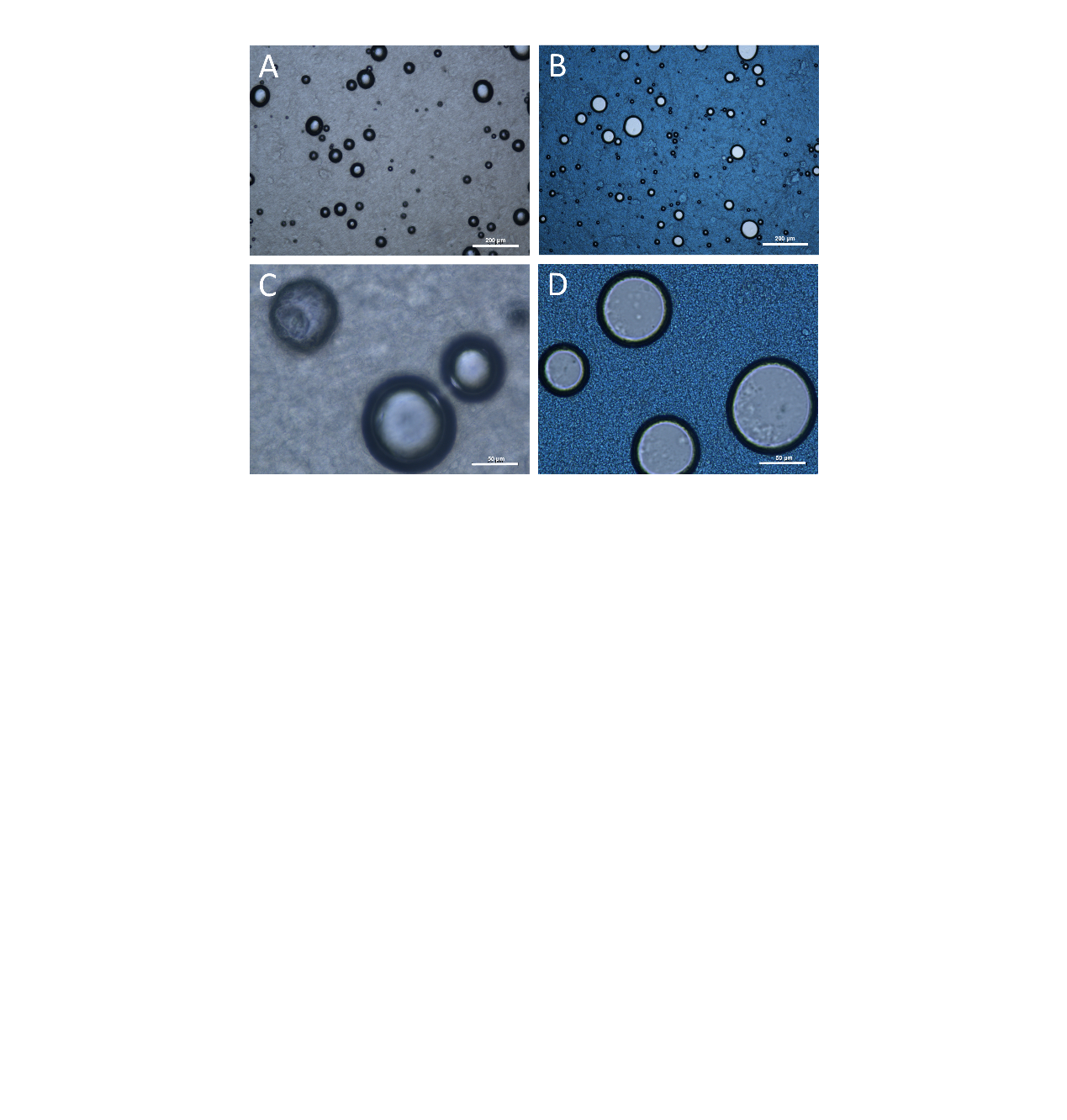
**

**Figure S2.** Effects of different concentrations (0.063, 0.125, 0.25, 0.5 mg/mL) of the antioxidant peptides (WP5, LW5 and YY6) on the viability of HDF-a cells irradiated by UVB. (ns, no significance, **P* < 0.05, ***P* < 0.01)


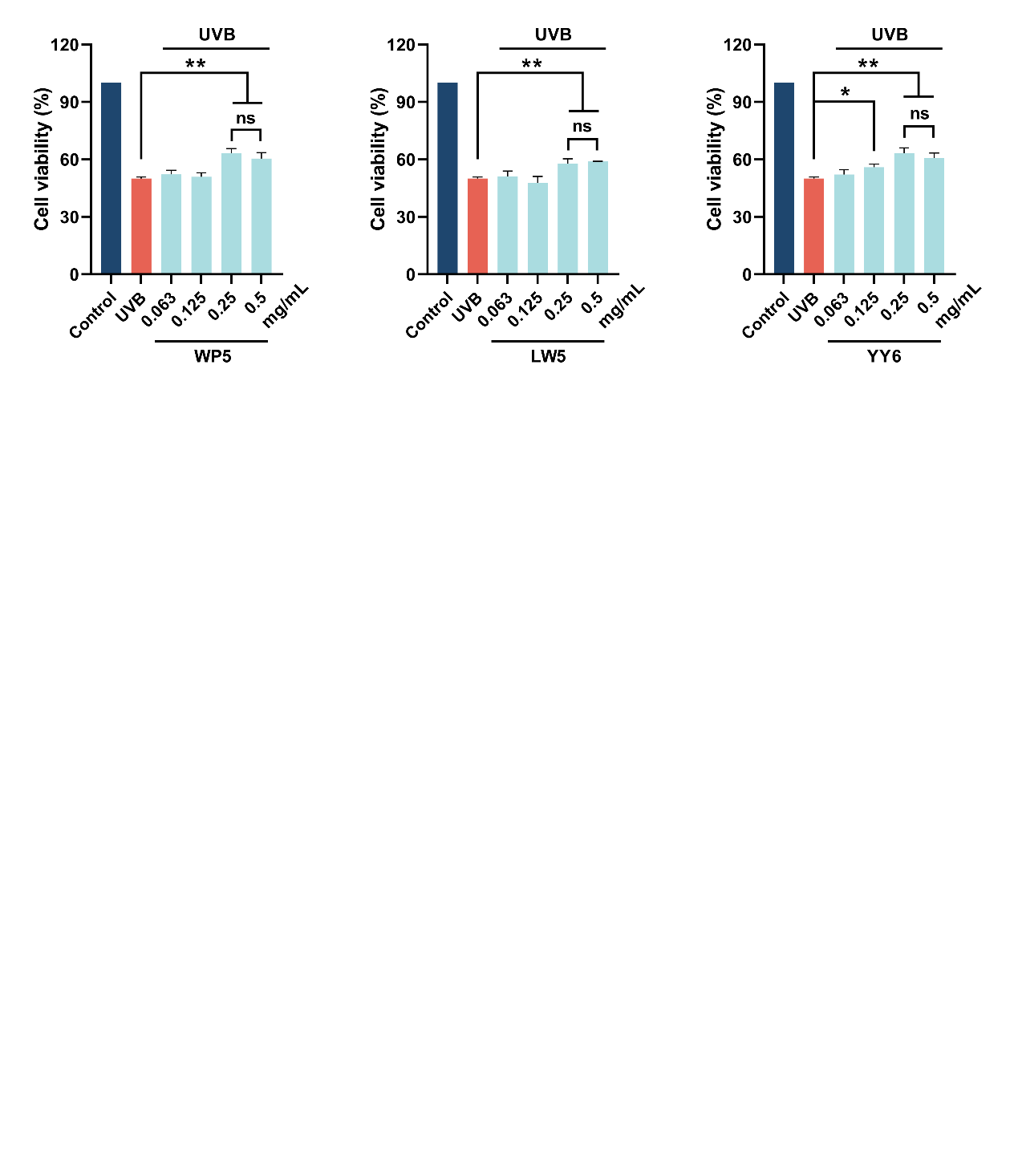

Supplement: Supplementary file 1 [file DataSheet1.docx]
